# Bacterial contamination, bacterial profile and antimicrobial susceptibility pattern of isolates from stethoscopes at Jimma University Specialized Hospital

**DOI:** 10.1186/1476-0711-12-39

**Published:** 2013-12-13

**Authors:** Teklu Shiferaw, Getenet Beyene, Tesfaye Kassa, Tsegaye Sewunet

**Affiliations:** 1 Department of Laboratory Science and Pathology, Jimma University, Jimma, Po.box -378, Ethiopia; 2 Department of microbiology, Adama hospital medical college, Adama, P.o. box-84, Ethiopia

## Abstract

**Introduction:**

Hospital acquired infections are recognized as critical public health problems. Infections are frequently caused by organisms residing in healthcare environment, including contaminated medical equipment like Stethoscopes.

**Objective:**

To determine bacterial contamination, bacterial profile and anti-microbial susceptibility pattern of the isolates from stethoscopes at Jimma University Specialized Hospital.

**Methodology:**

Cross-sectional study conducted from May to September 2011 at Jimma University Specialized Hospital. One hundred seventy-six stethoscopes owned by Health Care Workers (HCWs) and Medical students were randomly selected and studied. Self-administered structured questionnaire was used to collect socio-demographic data. Specimen was collected using moisten sterile cotton swab and 1 ml normal saline was used to transport the specimen, all laboratory investigations were done following standard microbiological techniques, at Microbiology Laboratory, Jimma University. SPSS windows version 16 used for data analysis and P <0.05 was considered statistically significant. Result: A total, of 151 (85.8%) stethoscopes were contaminated. A total of 256 bacterial strains and a mean of 1.44×10^4^ CFUs/diaphragm of stethoscopes was isolated. Of the 256 isolates, 133 (52%) were potential pathogens like S. aureus, Klebsiella spp., Citrobacter spp., Salmonella spp., Proteus spp., Enterobacter spp., P. aeruginosa and E. coli. All strains were resistant to multiple classes of antibiotics (two to eight classes of antibiotics). Disinfection practice was poor. Disinfection practice was found to be associated with bacterial contamination of stethoscopes (P < 0.05). High contamination rate 100 (90.9%) was observed among stethoscopes that had never been disinfected; while the least contamination 29 (72.2%) was found on those disinfected a week or less before the survey.

**Conclusion:**

Bacterial contamination of the stethoscope was significant. The isolates were potential pathogens and resistant to multiple classes of antibiotics. Stethoscope is potential vehicle in the transmission of infections between patients and Healthcare Workers. Stethoscope diaphragm should be disinfected before and after each patient contact.

## Introduction

Hospital environment is a reservoir of wide varieties of microorganisms. Several strains of pathogenic bacteria have been frequently reported colonizing medical equipments (like Stethoscopes) [1]. These pathogens include superbugs like Vancomycin Resistant *Enterococcus* spp., Methicillin Resistant and Sensitive *Staphylococcus* species and Multidrug resistant, *P. aeruginosa*, *E. coli*, *Klebsiella* spp. and *Streptococcus* spp. [2-4].

Medical equipments used in the non-critical care setting are less likely to have standard disinfection and cleaning protocols than equipments in the critical care setting. Thus medical care equipments are more likely to carry considerable number of pathogenic microorganisms [5]. The contamination of stethoscope particularly the diaphragm is reported mainly due to lack of regular disinfection (before and after examining each patient). A study from India reported that, 45% of general practitioners disinfect their stethoscope once a year or never and 35% disinfect their stethoscope monthly [1].

Infection prevention protocols are effective in reducing the health care associated infections [6]. The use of 70% propyl alcohol found to be effective in reducing contamination of stethoscopes and other medical equipments than other agents like detergents [6-9]. However, a study conducted by Hayden and his colleagues shows that, the implementation of such programs were hindered by poor compliance of Physicians, Nurses and other health care workers [10]. Inconvenience, time pressures, and skin damage from frequent washing are some of the reasons quoted by the health care personnel in that particular study [11]. A routine disinfection of stethoscope is hardly undertaken in most of the health care institutions worldwide [6,7,9].

During auscultation stethoscope contamination is common; if the same stethoscope is used for the next patient without disinfection, it might bring risk of infection to the patient and may continuously impose the risk serially to all patients [12]. Draping of stethoscopes around the neck is still a commonly seen practice, resulting in the risk of recontamination of the diaphragm of the stethoscope from the unclean earpieces, with normal flora and pathogenic bacterial strains harboring the ears of the HCWs.

A single stethoscope often used for all inpatients and outpatients [8,10]. The universal and unavoidable use of the stethoscope and its direct contact with multiple patients makes it an important potential factor in the dissemination of microorganisms from one patient to another.

Exposure of the already susceptible hospitalized patient to resident flora of the hospital environment (in most cases are multidrug resistant pathogens unless proved) may worsen the clinical condition of the patient. Periodic surveillance of medical equipments and hospital environments may help in identifying potential bacterial pathogens and associated factors. The aim of this study is to identify the contamination level of a stethoscope, bacterial profile, and antimicrobial susceptibility pattern of bacteria isolated from stethoscope diaphragms.

## Methodology

### Study area and period

The study was conducted at Jimma University Specialized Hospital (JUSH) from May to September 2011. Currently JUSH has 500 beds of ten wards and one ICU, serving a population of over fifteen million people in the southwest region. The hospital has a total number of 318 HCWs, of them 170 are clinicians (Nurses, Anesthetics and Doctors) and 616 are medical students; who are attached with both inpatient and outpatient services.

### Sample size and sampling technique

A total of 176 HCWs’ stethoscopes were considered for bacterial examination (8 from Nurses, 7 from Anesthetics, 57 form Doctors and 104 stethoscopes from medical students). Convenient sampling technique was used to select the stethoscope.

After getting informed consent from each participant, a pre structured questioner was used to collect data regarding the number of years in practice, gender of respondent, frequency of cleaning and type of disinfectant used to clean the stethoscope, and professional career. An identification number was assigned to each clinical site, and anonymity was maintained for all participants by substituting random numbers in place of names on each survey distributed.

### Specimen collection and identification of pathogen

Specimen was collected from the entire surface of the stethoscope diaphragm using moisten sterile cotton swab, with (0.9% w/v) physiological saline and inoculated on Blood agar, MacConkey agar and Chocolate agar plates (all culture media reagents were from Oxoid Ltd. Company, UK). The plates were incubated aerobically, except for Chocolate agar which was incubated in 5-10% CO_2_ concentrated candle jar, at 35°C for 24 hrs and observed for bacterial growth. Then aerobic gram-positive cocci contaminants initially were identified based on colony characterization, hemolysis pattern and gram staining of the colonies. Further identification was made with Catalase test, Mannitol fermentation, and Coagulase test.

For identification of Gram negative bacteria the following tests were done; catalase, oxidase, urease, indole, citrate utilization, lysine decarboxylation, glucose & lactose fermentation, gas & H_2_S production and motility tests. All biochemical test reagents were purchased from Oxoid Ltd. Company, UK.

Colony count ≥20 CFU/diaphragm was considered as significant contamination [13].

#### ***Antimicrobial susceptibility testing***

Antimicrobial susceptibility testing was carried out using disk diffusion method according to Clinical Laboratory Standards Institute (CLSI 2011) guide lines.

Discs for Gram positive bacteria contain the following antibiotics: - Cefoxitin (30 μg), Chloramphenicol (30 μg), Ciprofloxacin (5 μg), Clindamycin (2 μg), Erythromycin (15 μg), Gentamicin (10 μg), Penicillin (10 IU), and Tetracycline (30 μg), Trimethoprim-sulfamethoxazole (25 μg) and Vancomycin (30 μg). Discs for Gram positive bacteria contain the following antibiotics: - Ampicillin (10 μg), Cefotaxime (30 μg), Chloramphenicol (30 μg), Ciprofloxacin (5 μg), Gentamicin (10 μg), Nalidixic acid (30 μg), Norfloxacin (10 μg), Tetracycline (30 μg) and Trimethoprim-sulfamethoxazole (25 μg). All antibiotic discs were purchased from Oxoid Ltd. Company, UK and these drugs are commonly used in the study area. Cefoxitin (30 μg) disc was used for the detection of MRSA. The reference strains used as control were: *E. coli* (ATCC 25922), *S. aureus* (ATCC 25923) and *P. aeruginosa* (ATCC 27853).

Data were entered and analyzed using SPSS version 16.0 computer software. Comparisons were made using Chi-square test. P-value of <0.05 was considered indicative of a statistically significant difference.

Ethical clearance was secured from Ethical Clearance Committee of College of Public Health and Medical Sciences Jimma University. Permission was also obtained from Medical Director of Jimma University Specialized Hospital.

## Result

### Socio-demography

A total of 176 stethoscopes owned by nine different professionals were examined for bacterial contamination. The professionals include: - Clinical Specialists (6), Resident medical students (46), General Practitioners (5), Anesthetists (7), Nurses (8), Medical Interns (29), Clinical–II students (29), Clinical–I students (22) and Health Officer Interns (24). These health professionals were working in different eight wards namely: - Out Patient Department (37), Pediatrics ward (26), Medical ward (25), Surgical ward (23), Operating Room (22), Gynecology ward (20), Maternity ward (16), and ICU (7) (data is not shown).

### Bacterial contamination

**Figure 1:**
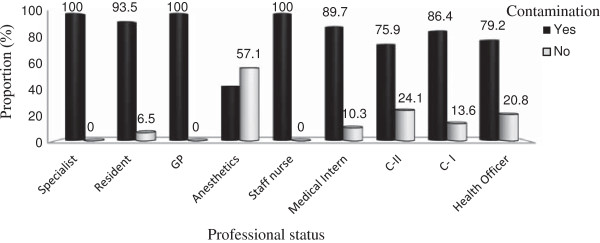
Rate of contamination versus professional status of the stethoscope owners at JUSH, May–Sep. 2011.

Of 176 stethoscopes examined, 151 (85.8%) were considerably contaminated (>20 CFUs/diaphragm), and the rest 25(14.2%) were not contaminated. All stethoscopes owned by Specialists, General Practitioners and Nurses were contaminated. The majority of stethoscopes of Resident students (93.5%), Medical Intern students (89.7%), and C-I students (86.4%) were contaminated. Relatively least contamination was observed on stethoscope diaphragms owned by Anesthetists (42.9%) (Figure 1).

**Figure 2:**
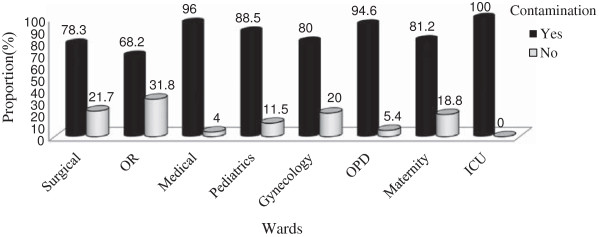
Rate of contamination versus stethoscope owners attending wards at JUSH, May–Sep. 2011.

The Frequency of contamination was 100% for stethoscopes from ICU, 96% for Medical ward, 94.6% for OPD. Almost all stethoscopes diaphragm collected from eight wards showed different degree of bacterial contaminations (Figure 2).

### Bacterial isolates

From 151 (85.8%) contaminated stethoscope diaphragms, a total of 256 bacterial strains were isolated. The maximum isolation per diaphragm was five species and the minimum was one bacterial species, with over all mean of 1.79 bacterial species per diaphragm.

Majority (52%) of the isolates were found to be potential pathogens. CoNS species was the most frequent isolate (40.2%) among gram-positive isolates; followed by *S. aureus* (30.9%) and Bacillus species (5.5%)*.*

From Gram negative isolates, *Klebsiella* spp*.* (4.7%) were the most common isolates, followed by *Citrobacter* spp. (4.3%), *Salmonella* spp*.* (3.5%)*, Proteus* spp*.* (3.5%)*, Enterobacter* spp. (3.1%), *P. aeruginosa* (1.2%) and *E. coli* (0.8%) (Table 1).

**Table 1:** Bacterial profile isolated from stethoscopes used at Jimma University Specialized Hospital from May-Sep2011

**Sr. No.**	**Isolated bacteria**	**Total no. (%)**
**1**	CoNS	103 (40.2)
**2**	*S. aureus*	79 (30.9)
**3**	*Bacillus* species	13(5.1)
**4**	*Klebsilla* species	12(4.7)
**5**	*Citrobacter* species	11 (4.3)
**6**	*Salmonella* species	9(3.5)
**7**	*Proteus* species	9 (3.5)
**8**	*Enterobacter* species	8 (3.1)
**9**	Gram positive filamentous	6 (2.3)
**10**	*P. aeruginosa*	3(1.2)
**11**	*E. coli*	2 (0.8)
**12**	*Micrococcus* species	1 (0.4%)
**13**	**Total isolates**	**256 (100%)**

Stethoscope from ICU ward harbored the highest (68.8%) potential pathogenic bacteria and least isolation for potential pathogenic bacteria (35.3%) was recorded from Maternity ward attendants’ stethoscope (Table 2).

**Table 2:** Pathogenicity of the bacterial isolates versus stethoscope owners among different wards at JUSH, May–Sep. 2011

**Pathogenicity**	**Wards**									
	**Surgical**	**OR**	**Medical**	**Pediatrics**	**Gynecology**	**OPD**	**Maternity**	**ICU**	**Isolates**	**p-value**
	**N** **o** **(%)**	**N** **o** **(%)**	**N** **o** **(%)**	**N** **o** **(%)**	**No** **(%)**	**N** **o** **(%)**	**N** **o** **(%)**	**N** **o** **(%)**	**N** **o** **(%)**	
**Potential**	13(39.4)	12(42.9)	26(66.7)	25(62.5)	11(47.8)	29(48.3)	6(35.3)	11(68.8)	**133(52)**	0.09
**Opportunistic**	20(60.6)	16(57.1)	13(33.3)	15(37.5)	12(52.2)	31(51.7)	11(64.7)	5(31.2)	**123(48)**	
**Total**	**33(100)**	**28(100)**	**39(100)**	**40(100)**	**23(100)**	**60(100)**	**17(100)**	**16(100)**	**256(100)**	

***Key***: % Percentage, No Number of isolates, OR Operating room, OPD Outpatient department, ICU Intensive care unit.

### Antimicrobial sensitivity pattern of the isolates

Over all 236 bacterial isolates were tested against fourteen different selected antibiotic discs. The antimicrobial drug resistance profile of bacterial isolates showed that, 26.6% of the *S. aureus* and 30.1% of CoNS isolates were Methicillin Resistant strains. All Methicillin Resistant strains were susceptible to Vancomycin. *S.aureus* and CoNS showed high resistance to Penicillin G (75.9% and 87.4% respectively). Relatively *S. aureus* (10.4%) and CoNS (9.7%) showed least resistance against Clindamycin (DA) (Table 3).

**Table 3:** Resistances patterns of the bacterial isolates form stethoscope diaphragms to commonly used antibiotic discs at JUSH, May–Sep. 2011

**Antibiotics tested**		**Resistances pattern of the isolates**								
	***S. aureus***	**CoNS**	***Klebsiella *** **spp** ***.***	***Citrobacter*** **spp** ***.***	***Salmonella *** **spp**	***Proteus *** **spp** ***.***	***Enterobacter *** **spp** ***.***	***P. aeruginos***	***E. coli***	**Total**
	**N** **o =** **79**	**N** **o =** **103**	**N** **o =** **12**	**N** **o =** **11**	***. *** **N** **o =** **9**	**N** **o =** **9**	**N** **o =** **8**	***a *** **N** **o =** **3**	**N** **o =** **2**	**236**
**AP(10 μg)**	-	-	8(66.7)	11(100)	9(100)	9(100)	5(62.5)	3(100)	1(50)	**46(**
**C(30 μg)**	35(44.3)	59(57.3)	6(50)	9(81.8)	8(88.9)	5(55.5)	3(37.5)	3(100)	0	**128**
**CIP(5 μg)**	14(17.7)	10(9.7)	0	3(27.3)	0	1(11.1)	3(37.5)	0	0	**31**
**CN(10 μg)**	21(26.6)	16(15.5)	5(41.6)	8(72.7)	9(100)	6(66.6)	3(37.5)	3(100)	0	**71**
**CTX(30 μg)**	-	-	9(75)	10(90.9)	9(100)	9(100)	6(75)	3(100)	1(50)	**47**
**DA (2 μg)**	8(10.4)	9(8.7)	-	-	-	-	-	-	-	**17**
**E (15 μg)**	22(27.8)	27(26.2)	-	-	-	-	-	-	-	**49**
**FOX(30 μg)**	21(26.6)	31(30.1)	-	-	-	-	-	-	-	**52**
**NA(30 μg)**	-	-	2(16.7)	3(27.3)	0	0	0	1(33.3)	1(50)	**7**
**NOR(10 μg)**	-	-	0	2(18.2)	0	1(11.1)	1(12.5)	0	0	**4**
**P(10 IU)**	60(75.9)	90(87.4)	-	-	-	-	-	-	-	**150**
**TE(30 μg)**	36(45.6)	33(41.8)	5(41.7)	7(63.6)	0	0	4(50)	3(100)	0	**88**
**TS(25 μg)**	29(36.2)	30(29.1)	5(41.7)	6(54.5)	0	0	3(37.5)	3(100)	0	**76**
**VA (30 μg)**	0	0	-	-	-	-	-	-	-	**0**

***Key***: Not done, 0 100 percent susceptible, μg Microgram, No Number, CoNS Coagulase negative staphylococci, AP Ampicillin, C Chloramphenicol, CIP Ciprofloxacin, CN Gentamicin, CTX Cefotaxime, DA Clindamycin, E Erythromycin, FOX Cefoxitin, NOR Norfloxacin, P Penicillin G, TS Trimethoprim-sulfamethoxazole/co-trimoxazole, TE Tetracycline, VA Vancomycin.

All *P. aeruginosa* isolates were resistant to Gentamicin, Cefotaxime, Trimethoprim-sulfamethoxazole, Tetracycline and Chloramphenicol. These isolates are susceptible to Ciprofloxacin and Norfloxacin. All *Salmonella* spp. showed resistance to Gentamicin, Cefotaxime and Ampicillin. However, Salmonella isolates were susceptible to Quinolones, Tetracycline and Trimethoprim-sulfamethoxazole. All *Proteus* spp., *Klebsiella* spp. and *E. coli* were susceptible to Ciprofloxacin, and showed highest resistances to Cefotaxime, with resistance rate of 100%, 75% and 50% respectively. All species of *Citrobacter* were resistant to Ampicillin and revealed least resistance to Nalidixic acid (NA) and Norfloxacin (Table 3).

Antibiogram of Gram positive bacterial isolates showed that 17.7% of S*. aureus* and 8.7% of CoNS isolates developed resistance to eight classes of antibiotics (Macrolides, Fluoro-quinolone, Cephalosporins, Aminoglycosides, Phenicols, Penicillins, Tetracyclines and Trimethoprim-sulfamethoxazole). Similarly gram-negative isolates showed MDR against two to eight drugs. Of the total nine isolates of *Salmonella* species one (11.1%) was resistant to three drugs and the rest eight isolates (88.9%) were resistant to four antibiotics (Table 4).

**Table 4:** Multidrug resistance patterns of bacterial isolates form stethoscope diaphragms to commonly used antibiotic classes at JUSH, May–Sep. 2011

**Bacteria**	**Quantity**	**Type of antibiotics**	**Isolates (Antibiotics Classes)**
**CoNS**	Max.	C,CIP,CN,DA,FOX,P,TE,TS	9(8)
	Min.	C,P	5(2)
***S. aureus***	Max.	C,CIP,CN,E,FOX,P,TE,TS	14(8)
	Min.	C,P	8(2)
***Klebsiella *** **spp.**	Max.	AP,C,CIP,CN,CTX,TE,TS	2(7)
	Min.	AP,CTX	1(2)
***Citrobacter *** **spp** ***.***	Max.	AP,C,CIP,CN,CTX,NA,TE,TS	8(7)
	Min.	AP,CTX	1(2)
***Salmonella *** **spp** ***.***	Max.	AP,C,CN,CTX	8(4)
	Min.	AP,CN,CTX	1(3)
***Proteus *** **spp.**	Max.	AP,C,CN,CTX	5(4)
	Min.	AP,CTX	1(2)
***Enterobacter *** **spp** ***.***	Max.	AP,C,CIP,CN,CTX,NOR,TE,TS	1(7)
	Min.	AP,CTX	2(2)
***P. aeruginosa***	Max.	AP,C,CN,CTX,NA,TE,TS	1(7)
	Min.	AP,C,CN,CTX,TE,TS	2(6)
***E. coli***	Max.	AP,CTX,NA	1(3)

***Key***: CoNS Coagulase negative staphylococci, AP Ampicillin, C Chloramphenicol, CIP Ciprofloxacin, CN Gentamicin, CTX Cefotaxime, DA Clindamycin, E Erythromycin, FOX Cefoxitin, NOR Norfloxacin, P Penicillin G, TE Tetracycline, TS Trimethoprim-sulfamethoxazole\Co-trimoxazole.

### Disinfection practice

Of the 176 stethoscopes studied, only 5 (2.8%) of the respondents (owners) reported that they disinfect their stethoscope, before and after examining each patient. Sixty-nine (95.8%) HCWs (Medical staffs and Residents), and 102 (98.1%) of medical students do not disinfect regularly but, the difference was not statistically significant (*p > 0.05*). All Doctors (Specialists, Residents and General Practitioners), Nurses, Medical Interns and Health Officers had reported that, they never disinfected their stethoscope diaphragms regularly.

All HCWs and medical students that do no disinfect stethoscopes regularly; 86 (50.3%) responded they have no perception about stethoscope disinfection and the rest reported lack of disinfectants.

## Discussion

The introduction of medical devices for management and treatment of diseases has contributed to the development of HAIs worldwide with the consequence that put the patient in to poor prognosis. The introduction of such devices is not wrong by itself, instead facilitates the medical procedures, but commitment deficit of the medical personnel’s to the infection prevention protocols was significant.

In the present study, almost all (97%) HCWs and Medical students do not follow the standard protocol set to prevent infections in using crucial medical equipment like stethoscopes. This finding is comparable with other previous studies that reported a rate of 97 to 100% [8,14-17]. On the other hand, Kilic and his colleagues reported relatively low rate of contamination of stethoscopes than this study [18]. There could be a variety of reasons for the differences. However, in the present study there is statistically significant difference between stethoscopes with no disinfection practice and regularly disinfected one’s (*p < 0.05*). This is in agreement with studies reported by Uneke and his colleagues (14,15).

A total of 85.8% stethoscopes were contaminated; which is consistent with previous studies reported by Zuliani-Maluf *et al*. (87%) [19]; Youngster *et al*. (85.7%) [3]; Uneke *et al.* (80.1%) (15), and Uneke *et al*. (79%) [15]. Whereas Marinella *et al*. [14] and wood *et al*. [4] reported 100% stethoscope contamination, which is higher than this finding. However, Africa-Purino and his colleagues found that, lower rate (57%) of contamination than the present study [20]. Furthermore; in this study the mean total bacterial count was 1.44 × 10^4^ CFUs/diaphragm. This is higher in comparison with both the standard [13] and with previous studies reported by Whittington *et al.* 34.5 CFUs/diaphragm [12], Wood *et al*. [4] 190.9 CFUs/diaphragm.

Two hundred fifty-six bacterial species were identified from 151 contaminated stethoscope diaphragms, with a mean of 1.79 species per diaphragm. The mean bacterial species count was lower as compared to (2.5 spp./diaphragm) reported by Miangi and Andriole [21], and higher than (1 spp./diaphragm) reported by Uneke and his colleagues [15]. Significant number of isolates in our findings is potential pathogens. As other similar studies, it can be possible to conclude that stethoscope diaphragms contamination with these microorganisms may spread leading causative agents of HAIs.

Gram-positive isolates (78.9%) were more frequent than gram-negative isolates (21.1%). This might be because of the direct contact of the stethoscope to human skin flora, which contains mostly gram-positive bacteria. Moreover, the lifespan of gram-negative bacteria is not more than six hours *in vitro*; the half-life span is less than an hour [22]. However, excessive bacterial colonization on stethoscope diaphragm enables them to remain alive for a longer period exceeding eight hours [2] whereas, gram-positive bacteria could remain alive for a longer period, even up to months [9,22].

One hundred thirty three (52%) isolates were potential pathogens, of them 40.6% were gram-negative isolates this is also higher than previous studies [1,2,16,18]. In addition, 53.7% were non-lactose fermenters, which are serious enteric pathogens. However; *S. aureus* 59.3% was the most common isolate over other potential pathogens isolated (*Klebsiella* spp*.*, *Citrobacter* spp*.*, *Salmonella* spp*.*, *Proteus* spp*., Enterobacter* spp*.*, *P. aeruginosa* and *E. coli*). Except *Salmonella* spp., all bacterial species were common isolates in both present and previous investigations of medical equipment and hospital environments [4,14-16].

In our investigation, we found a significant infestation of *Salmonella* spp. on nine consecutively surveyed stethoscope diaphragms of HCWs and medical students attending medical ward at the same survey time. In addition, the total colony count was beyond the upper limit; it is too many to count (TNTC) which is not in agreement with previous studies [2,11,14,21,23]. Excessive colonization on diaphragms might enable them to remain alive for a longer period. The same instance was evident in a study conducted by Youngster and his colleagues in Israel stated that “*At the time the study conducted there was an outbreak of Acinetobacter infections in Neonatal ICU; a stethoscope diaphragm that was positive for multidrug resistant Acinetobacter was collected from a resident during her rotation in the unit*” [3]. Such incidents may also show the potential danger of a stethoscope in the healthcare setting. The situation may be worse than expected when fueled with the ignorance of the medical professionals to the infection prevention protocols. The minimum survival time of most HAIs pathogenic organisms is about 2–18 hrs on the diaphragm surface of stethoscopes [22] and the clinicians spend on average less than 15 minutes with each patient, it is likely that stethoscope can serve as a vehicle for the spread of infection serially to the visiting patient in the hospital setting.

Although*, S. aureus* is a common flora of human skin; it is also well documented fact that *S. aureus* is a primary causative agent of HAI [24-27]. In addition, it was the most common pathogenic organism isolated from stethoscopes, with a prevalence of 4.2-54% regardless of the difference in setup and sample size in several studies [2,8,14-16,28]. The prevalence of methicillin resistance among the isolates was higher (MRSA 26.6% and MSSA 30.1%). Staphylococci isolates showed high resistance to commonly used β-lactam antibiotics (Penicillins 75%). This is similar to other previous studies reported elsewhere. *S. aureus* showed the least resistance to Ciprofloxacin in previous studies in the study area, the prevalence was rising from time to time 0% in 2007 [29] to 8% in 2011 [30], and in this study reported 17.7%.

All *P. aeruginosa* isolates were resistant to six most commonly used antibiotics in the study area, including Gentamicin, and Trimethoprim-sulfamethoxazole. All the *Salmonella* spp. isolates were resistant to Gentamicin, Cefotaxime and Ampicillin, and 88.9% to Chloramphenicol. About, 3/4 of the *Klebsiella*, *Citrobacter*, *Proteus* and *Enterobacter* species were showed the highest resistant to both Ampicillin and Cefotaxime. Except, *Citrobacter* and *Enterobacter* spp. all gram-negative bacteria isolates were susceptible to Ciprofloxacin, which was in-line with a study conducted by Uneke *et al*. [15,16] and Gebre-Sealssie [29]. Both gram positive and gram negative bateria have higher rates of resistance to different classes of antibitics; eight and seven classes of antibiotics respectively. Most of the antibiotic classes were used as treatment options in the study area. This might limit the therapeutic potions as the spread of these particular isolates goes on in this way and if intervention is not considered.

In our study, all licensed Doctors (Specialist, Resident and General Practitioner) reported they didn’t disinfect their stethoscope regularly. Hence, 98% had contaminated stethoscope diaphragms. This is consistent with the findings of Parmar *et al*. [8] and Wood *et al.*[4], in which none of the doctors disinfect their stethoscopes regularly. All Nurses had the same habit with their colleague doctors. However, in the previous other studies, Nurses reported to have had good thought than doctors [8,12,15]. This might be accounted to either the work burden or ignorance of the HCWs to adhere to infection prevention protocols. Like HCWs, 98.1% of Medical students reported they never disinfect the stethoscope before and after auscultating each patient. This is higher when compared to a study conducted by Uneke and his colleagues among Nigerian Medical student which reported 91% [16]. Of HCWs and Medical students attending Medical, Pediatrics, Gynecology, OPD and ICU wards, none of them reported to disinfect their stethoscope regularly. However, only 13.6% from OR and 4.3% from Surgical ward attendants reported that they disinfect their stethoscopes regularly before and after seeing each patient.

About 55% HCWs and Medical students, attending critical care areas like; ICU, OR, Surgical and Maternity wards, reported that, they have no perception about disinfection of the stethoscope. Twenty-four (31.9%) of HCWs and 5.9% Medical students reported lack of adequate and appropriate disinfectant.

Sufficient emphasis on disinfection practices of such unavoidable medical equipment for patient care is mandatory. Lack of focus in the medical curriculum might be the possible reason for the lack of awareness and high degree of contamination. This was also indicated by several other investigators. Similarly our study indicated high contamination rate of stethoscopes with potential pathogens that may cause variety of diseases. These bacterial strains are resistant to commonly used antimicrobial agents. Therefore strict adherence to stethoscope disinfection, and also to infection prevention protocols for possible other medical equipments may minimize hospital acquired infections and ensure improved patient safety in hospital environment.

## Conclusion

Rate of contamination recorded in this study was higher than any set standard. It is also greater as compared to previous studies conducted elsewhere. Most of the strains isolated were potential pathogen and known causes of hospital acquired infections. Furthermore these isolates are resistant to multiple classes of antimicrobial agents prescribed in the hospital. Most of the health care workers reported they have no perception about stethoscope disinfection. Therefore; there should be strict and careful handling of stethoscopes. Otherwise it could be a potential vehicle for transmission of hospital acquired infections.

## Competing interest

The authors declare that they have no competing interest.

## Authors’ contribution

All authors have made substantial contribution in (1) the conception and design of the study; acquisition of data or analysis and interpretation of the data, (2) drafting the article or revising it critically for important intellectual content, (3) final approval of the version to be submitted, and (4) agreed to be accountable for all aspects of the work in ensuring that questions related to the accuracy or integrity of any part of the work are appropriately investigated and resolved.
